# Prevalence and Genomic Characteristics of *mcr*-Positive Escherichia coli Strains Isolated from Humans, Pigs, and Foods in China

**DOI:** 10.1128/spectrum.04569-22

**Published:** 2023-04-12

**Authors:** Xiaoyu Lu, Pei Zhang, Pengcheng Du, Xiuli Zhang, Juan Wang, Yingying Yang, Honghu Sun, Zhiqiang Wang, Shenghui Cui, Ruichao Li, Li Bai

**Affiliations:** a Jiangsu Co-Innovation Center for Prevention and Control of Important Animal Infectious Diseases and Zoonoses, College of Veterinary Medicine, Yangzhou University, Yangzhou, Jiangsu, China; b Key Laboratory of Food Safety Risk Assessment, National Health Commission of the People’s Republic of China, China National Center for Food Safety Risk Assessment, Beijing, China; c Center for Disease Control and Prevention of Henan Province, Zhengzhou, China; d Institute of Infectious Diseases, Beijing Ditan Hospital, Capital Medical University, Beijing, China; e Beijing Key Laboratory of Emerging Infectious Diseases, Beijing, China; f College of Veterinary Medicine, Northwest A&F University, Yangling, Shaanxi, China; g Department of Neurology, Gaotang County People's Hospital, Gaotang, Shandong, China; h Chengdu Institute for Food and Drug Control, Chengdu, China; i Institute of Comparative Medicine, Yangzhou University, Yangzhou, Jiangsu, China; j Department of Food Science, National Institutes for Food and Drug Control, Beijing, China; Brown University

**Keywords:** *Escherichia coli*, polymyxin B, *mcr-1*, humans, pigs, foods

## Abstract

Colistin is one of the last-resort antibiotics for treating infections caused by multidrug-resistant (MDR) Gram-negative bacteria. However, *mcr* genes conferring resistance to colistin have been widely identified, which is considered a global threat to public health. Here, we investigated the prevalence and characteristics of *mcr*-harboring Escherichia coli strains isolated from humans, animals, and foods in China by PCR, antimicrobial susceptibility testing, conjugation experiments, molecular typing, genome sequencing, and bioinformatics analysis. In total, 135 *mcr-1*-harboring E. coli isolates were acquired from 847 samples, and 6 isolates carried *mcr-3*. Among them, 131 isolates were MDR bacteria. Sixty-five resistance genes conferring resistance to multiple antimicrobials were identified in 135 isolates. The diverse pulsed-field gel electrophoresis (PFGE) patterns and sequence types (STs) of *mcr-1*-carrying isolates demonstrated that clonal dissemination was not the dominant mode of *mcr-1* transmission. Seven types of plasmids were able to carry *mcr-1* in this study, including IncI2, IncX4, IncHI2, p0111, IncY, and two hybrid plasmids. The genetic structures carrying *mcr-1* of 60 isolates were successfully transferred into the recipient, including 25 IncI2 plasmids, 23 IncX4 plasmids, and an IncHI2 plasmid. *mcr-1–pap2* was the dominant *mcr-1*-bearing structure, followed by IS*Apl1–mcr-1–pap2–*IS*Apl1* (Tn*6330*) and IS*Apl1–mcr-1–pap2*, among 7 *mcr-1*-bearing structures of 135 isolates. In conclusion, IncI2, IncX4, and IncHI2 plasmids were the major vectors spreading *mcr-1* from different geographical locations and sources. The prevalence of Tn*6330* may accelerate the transmission of *mcr-1*. Continuous surveillance of *mcr-1* and variants in bacteria is vital for evaluating the public health risk posed by *mcr* genes.

**IMPORTANCE** The spread of polymyxin-resistant Enterobacteriaceae poses a significant threat to public health and challenges the therapeutic options for treating infections on a global level. In this study, *mcr-1*-bearing ST10 E. coli was isolated from pigs, pork, and humans simultaneously, which demonstrated that ST10 E. coli was an important vehicle for the spread of *mcr-1* among animals, foods, and humans. The high prevalence of *mcr-1*-positive E. coli strains in pigs and pork and the horizontal transmission of *mcr-1*-bearing plasmids in diverse E. coli strains suggest that pigs and pork are important sources of *mcr-1*-positive strains in humans and pose a potential threat to public health. Additional research on the prevalence and characteristics of *mcr-1*-positive E. coli is still required to facilitate early warning to improve polymyxin management in hospitals.

## INTRODUCTION

The spread of antimicrobial-resistant bacteria among humans, animals, and the environment poses a global threat to public health. A recent systematic analysis estimated that nearly 4.95 million deaths were associated with bacterial antimicrobial resistance (AMR) worldwide in 2019, with 1.27 million of these deaths being directly attributable to bacterial AMR (1). Polymyxins (polymyxin B and colistin) have been reintroduced as crucial last-resort antibiotics to treat infections caused by multidrug-resistant (MDR) Gram-negative bacteria (2, 3). Colistin has been commonly used to treat infections in animals and as a growth promoter in animal feeds, which contributed to the large-scale resistance of bacteria to colistin. The plasmid-borne mobile colistin resistance gene *mcr-1* was first characterized in food-producing animals and humans in China in late 2015 (4), and colistin resistance owing to *mcr-1* has attracted much public attention due to the rapid spread of plasmids by horizontal transfer. Since then, nine novel variants of *mcr* genes (*mcr-2* to *mcr-10*) have been identified worldwide (5, 6).

The *mcr-1* gene is still the most extensively disseminated gene of the *mcr* family, and the prevalence of *mcr-1*-positive isolates has been described in more than 50 countries across 6 continents over the past few years (7, 8). IncI2, IncX4, and IncHI2 plasmids are major vectors that remarkably facilitated the widespread dissemination of *mcr-1*. In addition to plasmid-borne transmission, *mcr-1* has also been identified on the chromosome (9). Importantly, these *mcr-1*-carrying bacteria have efficiently disseminated not only to animals, including livestock, companion animals, insects, and wildlife (10–13), but also to humans and the environment (4, 14). Notably, significantly varying levels of *mcr-1* carriage were observed in different Escherichia coli isolates, which was obviously associated with their origin (7). It is extremely possible that *mcr*-positive bacteria can spread from animals and the environment to humans, thus endangering public health.

Due to the rapid spread of *mcr*-positive isolates among humans, animals, and the environment, the Chinese government banned the use of polymyxin as an animal growth promoter on 1 May 2017 (15). A previous study showed that the withdrawal of polymyxin from animal feed had a significant effect on reducing colistin resistance in humans and animals in China (16, 17). In order to provide more information for further studies on the effect of the polymyxin withdrawal policy on the prevalence of polymyxin-resistant bacteria, research on the dissemination of *mcr*-positive E. coli before 2017 from humans, animals, and foods is of great significance. In this study, we collected 847 samples, including pig feces, retail foods, and patient feces, from Hebei, Henan, and Sichuan Provinces from July to December 2016 to understand the prevalence and characteristics of *mcr*-positive E. coli strains from different sources before 2017 in China.

## RESULTS

### Identification of *mcr*-positive isolates.

A total of 847 samples of pig feces (*n* = 99), retail foods (including 155 pork samples, 66 chicken meat samples, and 226 vegetable samples), and patient feces (*n* = 301) were collected from Hebei, Henan, and Sichuan Provinces in China from July to December 2016. Among these samples, 135 *mcr-1*-positive nonduplicated E. coli isolates were identified (135/847; 15.9%), including 61 isolates from 447 retail food samples, 54 isolates from 99 pig fecal samples, and 20 isolates from 301 patient fecal samples (Table 1). Among these *mcr-1*-bearing E. coli isolates, 6 isolates (CP8-3, CP53, CP55, CP61, CP66-6, and CP131) also carried the *mcr-3* gene, and we investigated them in previous studies (18, 19). The prevalence of *mcr-1*-positive E. coli was the highest in pig feces (54/99; 54.6%), whereas that in patient feces (20/301; 6.6%) was lower than that in the other samples. Among 447 retail food samples, the prevalence of *mcr-1*-positive E. coli strains isolated from pork was the highest (51/155; 32.9%), and the prevalence in vegetable samples was the lowest (4/226; 1.8%). The positivity rate for *mcr-1*-carrying E. coli isolates was 9.1% (6/66) in chicken meat samples (Table 1). Notably, among 135 *mcr-1*-bearing E. coli isolates, 38 isolates originated from the 285 samples from Hebei Province (38/285; 13.3%), 16 isolates originated from the 208 samples from Henan Province (16/208; 7.7%), and 81 isolates originated from the 354 samples from Sichuan Province (81/354; 22.9%). *mcr-1*-carrying E. coli isolates were the most prevalent in Sichuan (Table 1).

**TABLE 1:** Numbers of samples and *mcr-1*-positive E. coli isolates from Hebei, Henan, and Sichuan Provinces in China investigated in this study

Province	No. (%) of samples of origin						No. (%) of *mcr-1*-positive E. coli isolates
	Pork	Chicken	Vegetables	Pig feces	Patient feces	Total	
Hebei	80		31		174	285	38 (13.3)
Henan	32	61	60		55	208	16 (7.7)
Sichuan	43	5	135	99	72	354	81 (22.9)

Total	155	66	226	99	301	847	135 (15.9)

*mcr-1*-positive E. coli	51 (32.9)	6 (9.1)	4 (1.8)	54 (54.6)	20 (6.6)	135 (15.9)	

### Resistance phenotypes.

Antimicrobial susceptibility testing revealed that the MICs of polymyxin B for 135 *mcr-1*-bearing isolates ranged from 4 to 16 μg/mL. Among them, 131 isolates (131/135; 97.0%) were MDR bacteria conferring resistance to 3 or more classes of antimicrobials (Table 2). Worryingly, 23 isolates (23/135; 17.0%) were resistant to 6 classes of antimicrobials, 26 isolates (26/135; 19.3%) were resistant to 7 classes of antimicrobials, 36 isolates (26.7%) were resistant to 8 classes of antimicrobials, and 22 isolates (16.3%) showed resistance to 9 classes of antimicrobials (Table 2). In addition, these isolates exhibited the highest rates of resistance to tetracycline (130/135; 96.3%), followed by ampicillin (120/135; 88.9%), chloramphenicol (119/135; 88.1%), trimethoprim-sulfamethoxazole (117/135; 86.7%), ciprofloxacin (93/135; 68.9%), and cefotaxime (50/135; 37.0%) (Table 3).

**TABLE 2:** Antimicrobial resistance profiles and corresponding numbers of *mcr-1*-positive E. coli isolates^*a*^

Antimicrobial resistance profile	No. of antimicrobial classes	No. of isolates	No. of isolates with the same no. of antimicrobial classes (%)
PB	1	1	1 (0.7)
TET-PB	2	3	3 (2.2)
TET-AMP-PB	3	1	2 (1.4)
TET-SXT-PB	3	1	
TET-AMP-CHL-PB	4	2	9 (6.7)
TET-AMP-CHL-PB	4	1	
TET-AMP-SXT-PB	4	1	
TET-CHL-SXT-PB	4	3	
TET-CHL-FLU-PB	4	1	
TET-FLU-SXT-PB	4	1	
TET-AMP-CHL-FLU-PB	5	1	13 (9.6)
TET-AMP-CHL-SXT-PB	5	2	
TET-AMP-FLU-SXT-PB	5	1	
TET-CHL-SXT-NAL-PB	5	3	
TET-GEN-AMP-CHL-PB	5	1	
AMP-CHL-CIP-AZI-PB	5	2	
TET-AMP-CHL-FLU-PB	5	1	
TET-CHL-FLU-SXT-PB	5	1	
AMP-FLU-SXT-AZI-PB	5	1	
CEP-TET-AMP-CHL-SXT-PB	6	1	23 (17.0)
CEP-TET-AMP-FLU-SXT-PB	6	1	
TET-AMP-CHL-FLU-SXT-PB	6	11	
TET-AMP-CHL-SXT-AZI-PB	6	3	
TET-CHL-FLU-SXT-AZI-PB	6	1	
TET-GEN-AMP-CHL-AZI-PB	6	5	
TET-GEN-AMP-CHL-SXT-PB	6	1	
CEP-AMP-CHL-SXT-FLU-AZI-PB	7	1	26 (19.3)
CEP-GEN-AMP-CHL-SXT-FLU-PB	7	1	
CEP-TET-AMP-CHL-FLU-SXT-PB	7	5	
TET-AMP-CHL-FLU-SXT-AZI-PB	7	3	
TET-GEN-AMP-CHL-SXT-AZI-PB	7	6	
TET-GEN-AMP-CHL-SXT-FLU-PB	7	7	
TET-GEN-AMP-FLU-SXT-AZI-PB	7	3	
TET-GEN-AMP-CHL-FLU-SXT-AZI-PB	8	18	36 (26.7)
CEP-TET-AMP-CHL-SXT-FLU-AZI-PB	8	8	
CEP-TET-GEN-AMP-CHL-SXT-AZI-PB	8	2	
CEP-TET-GEN-AMP-CHL-FLU-SXT-PB	8	8	
CEP-TET-GEN-AMP-CHL-FLU-SXT-AZI-PB	9	22	22 (16.3)

a PB, polymyxin B; TET, tetracycline; AMP, ampicillin; SXT, trimethoprim-sulfamethoxazole; CHL, chloramphenicol; FLU, resistance to both ciprofloxacin and nalidixic acid; NAL, nalidixic acid; GEN, gentamicin; CIP, ciprofloxacin; AZI, azithromycin; CEP, resistance to both cefotaxime and ceftazidime.

**TABLE 3:** Resistance phenotypes of 135 *mcr-1*-bearing E. coli isolates from different origins

Antimicrobial	No. (%) of isolates resistant to antimicrobials			
	Food (*n* = 61)	Pig feces (*n* = 54)	Patient feces (*n* = 20)	Total
Ampicillin	54 (88.5)	51 (94.4)	15 (75.0)	120 (88.9)
Cefotaxime	31 (50.8)	7 (13.0)	12 (60.0)	50 (37.0)
Ceftazidime	12 (19.7)	4 (7.4)	6 (30.0)	22 (16.3)
Imipenem	0 (0.0)	0 (0.0)	0 (0.0)	0 (0.0)
Gentamicin	27 (44.3)	37 (68.5)	10 (50.0)	74 (54.8)
Trimethoprim-sulfamethoxazole	54 (88.5)	46 (85.2)	17 (85.0)	117 (86.7)
Chloramphenicol	56 (91.8)	48 (88.9)	15 (75.0)	119 (88.1)
Azithromycin	31 (50.8)	33 (61.1)	10 (50.0)	74 (54.8)
Tetracycline	58 (95.1)	54 (100.0)	18 (90.0)	130 (96.3)
Ciprofloxacin	49 (80.3)	32 (59.3)	12 (60.0)	93 (68.9)
Nalidixic acid	38 (62.3)	20 (37.0)	14 (70.0)	72 (53.3)
Polymyxin B	61 (100.0)	54 (100.0)	20 (100.0)	135 (100.0)

### Molecular typing.

**FIG 1:**
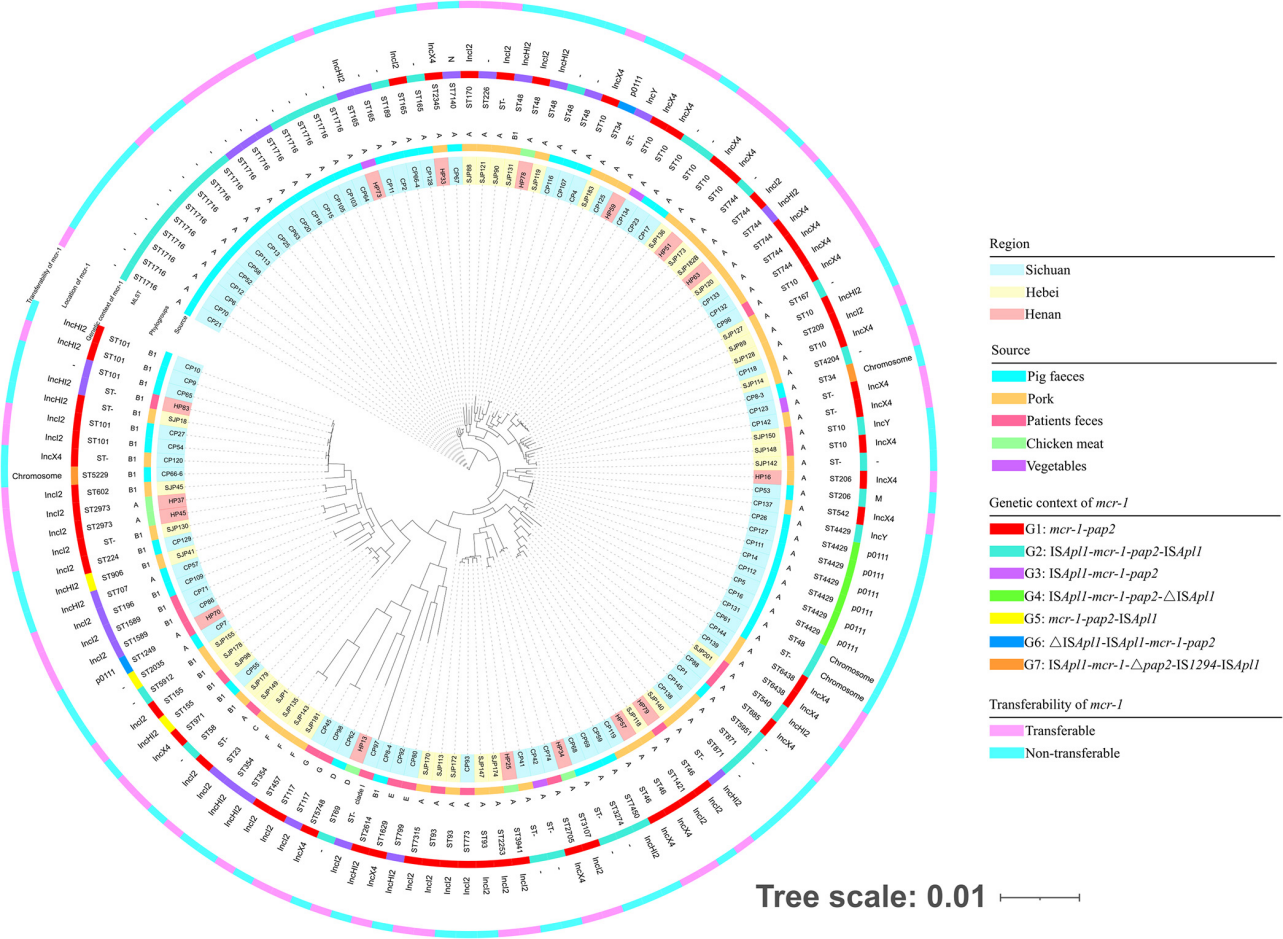
Phylogenetic tree of 135 *mcr-1*-positive E. coli isolates from different sources and their basic characterization. Strain identifiers with differently colored backgrounds correspond to regions of sampling. In the “Location of *mcr-1*” column, “N” stands for IncHI1A-IncHI1B(R27), and “M” indicates IncN-IncHI1B(R27)-IncHI1A-IncFIA(HI1).

The vast majority of isolates were distributed on different genetic branches according to the pulsed-field gel electrophoresis (PFGE) patterns, and the genetic relationships were distant. Therefore, there was no absolute dominant PFGE spectrum (see Fig. S1 in the supplemental material). In addition, some *mcr-1*-positive E. coli isolates exhibited genetically similar PFGE types with 100% homology, such as CP9 and CP10, CP18 and CP20, CP103 and CP113, as well as CP111 and CP112 (Fig. S1), all of which were obtained from pig feces, implying that the clonal spread of *mcr-1*-positive E. coli strains occurred among farmed pigs. The E. coli isolates of the present study were classified using Clermont typing, and the majority belonged to group A (101/135; 74.8%), followed by group B1 (23/135; 17.0%). Groups C, D, E, F, and G were also observed in the present study (Fig. 1). The vast majority of isolates from Sichuan clustered into two distinct subclades in the phylogenetic tree based on single-nucleotide polymorphisms (SNPs) of core genomes (Fig. 1). The remaining isolates were scattered across various branches of the phylogenetic tree (Fig. 1). The phylogenetic tree revealed pronounced genotypic diversity among *mcr-1*-bearing isolates, and clonal spread occurred in samples from the same region and source but was not the dominant mode of *mcr-1* transmission (Fig. 1).

**FIG 2:**
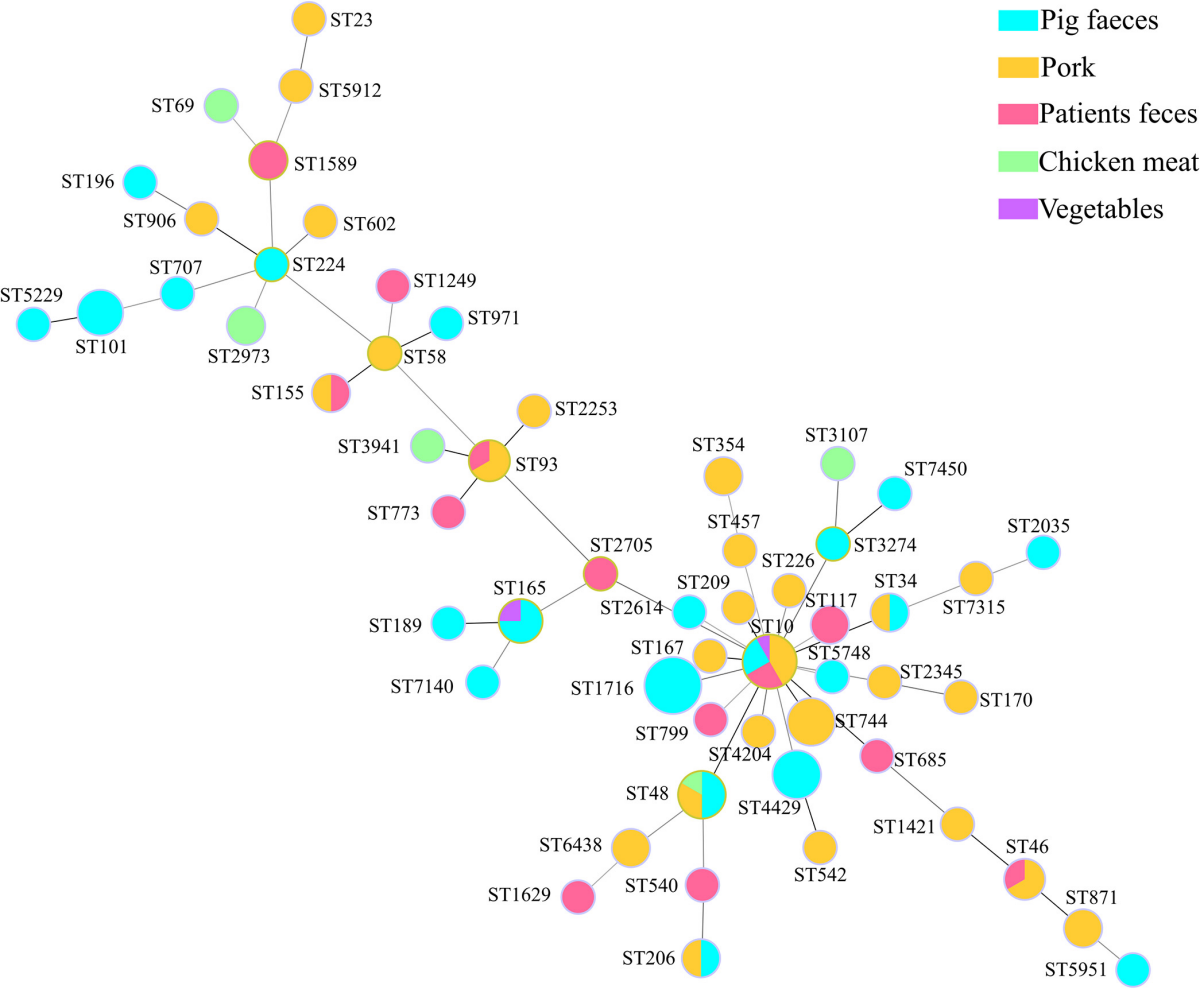
Minimum-spanning tree of *mcr-1*-carrying E. coli isolates by MLST type. Each circle represents an ST, and different colors represent different sources of *mcr-1*-carrying isolates. Sixteen untyped isolates are not shown.

Multilocus sequence typing (MLST) showed that 119 *mcr-1*-positive E. coli isolates belonged to 57 distinct sequence types (STs), and the remaining 16 isolates belonged to unknown STs (Fig. 1 and 2). Among the known STs, ST1716 (11.9%; 16/135), ST10 (8.9%; 12/135), ST4429 (5.2%; 7/135), ST744 (4.4%; 6/135), ST48 (4.4%; 6/135), and ST101 (3.7%; 5/135) were determined as the predominant STs (Fig. 2). Notably, identical STs, including ST10, ST34, ST48, and ST206, were found from pig feces and pork samples. Identical STs, including ST10, ST46, ST93, and ST155, were observed in patient feces and pork (Fig. 2). This demonstrated that ST10 E. coli was an important vehicle for the spread of *mcr-1* among animals, foods, and humans. In addition, 72 *mcr-1*-harboring E. coli isolates obtained from Sichuan were classified into 33 STs (the remaining 9 isolates belonged to unknown STs), whereas 32 *mcr-1*-positive E. coli isolates recovered from Hebei were divided into 22 STs (the remaining 6 isolates belonged to unknown STs), and 15 *mcr-1*-bearing E. coli isolates from Henan were separated into 12 STs (the 1 remaining isolate belonged to an unknown ST) (Fig. 1 and 2). The distribution of different STs in different regions indicated the genetic diversity of *mcr-1*-positive E. coli isolates. The diverse PFGE patterns and STs of *mcr-1*-carrying isolates demonstrated that clonal dissemination was not the dominant mode of *mcr-1* transmission.

### Prevalence of acquired antibiotic resistance genes.

**FIG 3:**
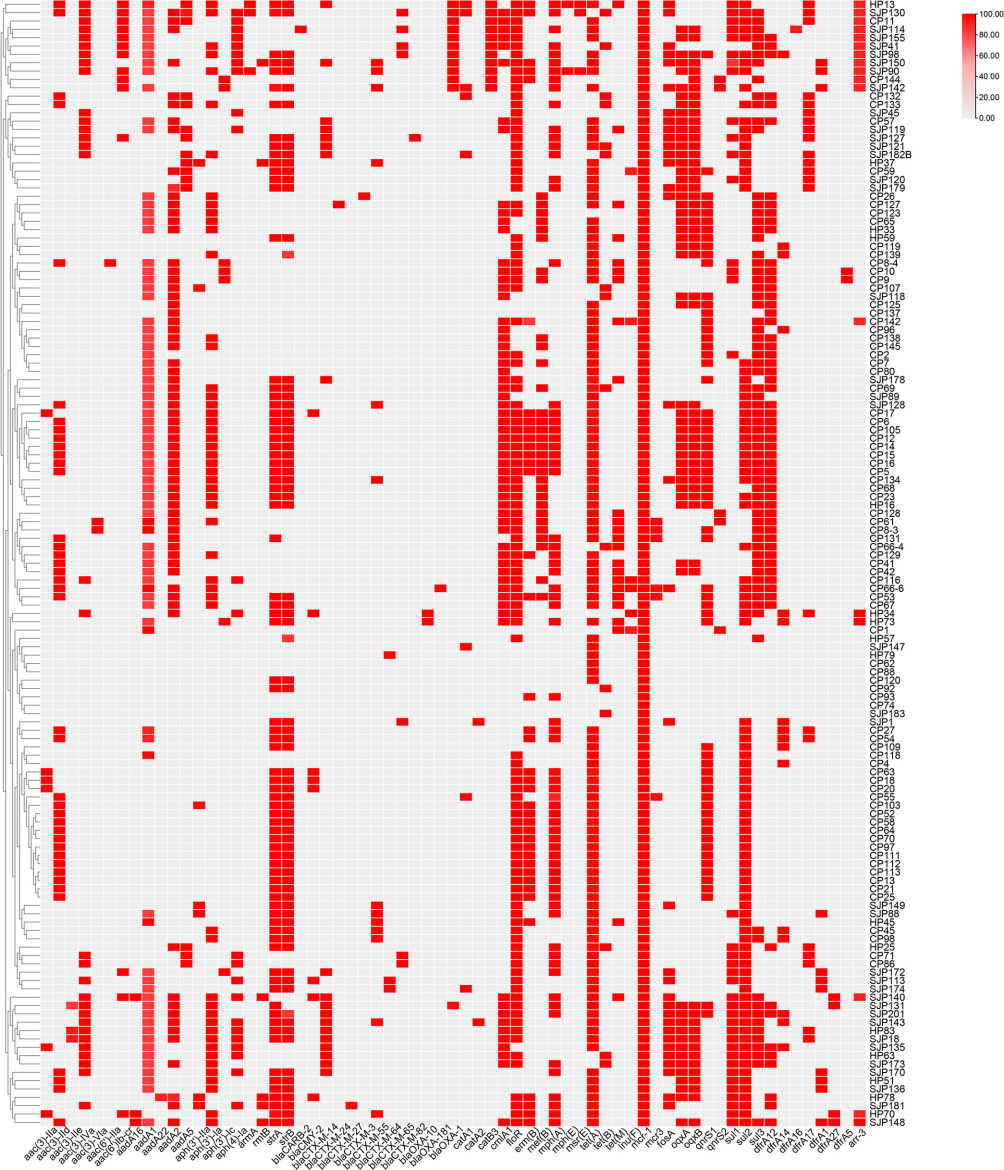
Distribution of antimicrobial resistance genes in 135 *mcr-1*-positive E. coli isolates. The red rectangles indicate the presence of antimicrobial resistance genes.

The acquired antibiotic resistance genes in 135 *mcr-1*-positive E. coli isolates displayed significant variations. A total of 65 resistance genes conferring resistance to diverse antimicrobials were identified in these isolates (Fig. 3). The most prevalent resistance genes were *tet*(A), conferring tetracycline resistance (115/135; 85.19%); *floR*, conferring florfenicol resistance (106/135; 78.52%); and *sul2*, conferring sulfonamide resistance (92/135; 68.15%). Apart from *tet*(A), *floR*, and *sul2*, 2 resistance genes responsible for tetracycline resistance, 4 resistance genes responsible for phenicol resistance, and 2 resistance genes responsible for sulfonamide resistance were observed. A total of 20 aminoglycoside resistance genes were detected, among which *aadA1* (79/135; 58.52%), *strA* (79/135; 58.52%), and *strB* (79/135; 58.52%) were the most prevalent. It is worth noting that each of the 126 isolates contained at least one aminoglycoside resistance gene. In addition, 13 β-lactamase-encoding genes were detected, among which *bla*_CTX-M-14_ (19/135; 14.07%) was the most prevalent. Furthermore, 7 trimethoprim resistance genes, 4 fluoroquinolone resistance genes, and 5 macrolide resistance genes were identified. One fosfomycin resistance gene, *fosA*; one lincosamide resistance gene, *lnu*(F); and one rifampin resistance gene, *arr-3*, were identified (Fig. 3).

### Characteristics of *mcr-1*-bearing plasmids.

Conjugation experiments with 135 *mcr-1*-carrying isolates were performed to determine the transmissibility of *mcr-1*. The genetic structures carrying the *mcr-1* genes of 60 E. coli isolates with polymyxin B resistance phenotypes were successfully transferred into the recipient E. coli strain 26R 793 (Fig. 1). Using draft sequences, we performed a comprehensive analysis of the location of *mcr-1*. Among *mcr-1*-carrying isolates from Hebei, *mcr-1* in 17 isolates was transferred to the recipients, and the *mcr-1* gene existed mainly on IncI2 plasmids (*n* = 12), followed by IncX4 plasmids (*n* = 2) (Fig. 1 and Table S1). Among *mcr-1*-carrying isolates from Henan, *mcr-1* in 10 isolates was transferred, and the *mcr-1* gene was located on the IncI2 (*n* = 5) and IncX4 (*n* = 5) plasmids (Fig. 1 and Table S1). Among *mcr-1*-carrying isolates from Sichuan, *mcr-1* in 33 isolates was transferred, and the *mcr-1* gene was mediated mainly by IncX4 (*n* = 16) and IncI2 (*n* = 8) plasmids, followed by IncHI2 plasmids (*n* = 1) (Fig. 1 and Table S1). These results showed that IncI2 and IncX4 plasmids were the dominant vehicles responsible for disseminating the *mcr-1* gene in different geographical locations and sources through horizontal transfer. However, whether the most dominant vector was IncI2 or IncX4 plasmids varied by region.

By analyzing the draft assembly sequences, we inferred 7 types of plasmids carrying *mcr-1* in 95 isolates, including IncI2 (*n* = 33), IncX4 (*n* = 26), IncHI2 (*n* = 19), p0111 (*n* = 8), IncY (*n* = 3), and the hybrid plasmid types IncHI1A-IncHI1B(R27) (*n* = 1) and IncN-IncHI1B(R27)-IncHI1A-IncFIA(HI1) (*n* = 1) (Fig. 1). The *mcr-1* gene in 4 isolates coharboring *mcr-1* and *mcr-3* was located on the chromosome (19). However, the locations of *mcr-1* in the remaining 40 isolates could not be determined by draft sequences due to the short contigs (<8 kb) and/or no replicon within the contig (Fig. 1). We found that *mcr-1* was mediated primarily by IncI2, IncX4, and IncHI2 plasmids in this study, which was consistent with the results of previous studies (10, 12, 20).

**FIG 4:**
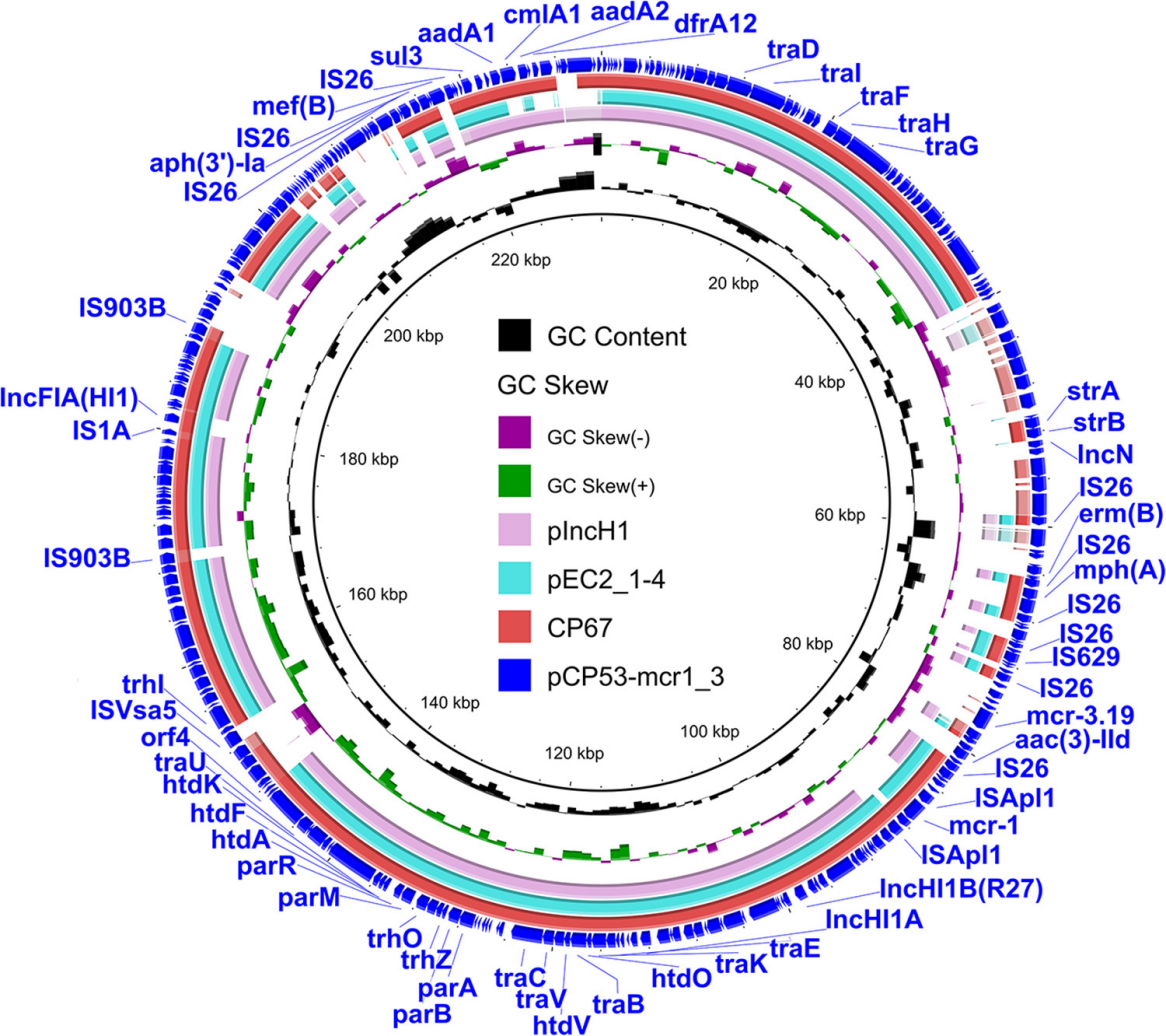
Circular comparison of hybrid plasmids. The outermost circle denotes the reference plasmid pCP53-mcr1_3 with annotated genes. The red circle indicates the draft genome assemblies of E. coli CP67. Plasmids pEC2_1-4 and pIncH1 were retrieved from the NCBI database.

Two *mcr-1*-bearing hybrid plasmids were found in this study. The *mcr-1*-bearing hybrid plasmid pCP53-mcr1_3 [with multiple replicons, including IncN, IncHI1B(R27), IncHI1A, and IncFIA(HI1)] in E. coli CP53 also carried the *mcr-3* gene (Fig. 4), and we characterized this plasmid previously (18). In the draft sequence of E. coli CP67, *mcr-1* and IncHI1A-IncHI1B(R27) replicons were located within the same contig. The BLASTn alignment indicated that the *mcr-1*-carrying contig showed 99.79% identity (94% coverage) to the sequence of plasmid pEC2_1-4 (GenBank accession number CP016183) of E. coli EC2_1. pEC2_1-4 was an *mcr-1*-bearing plasmid with IncHI1B(R27), IncHI1A, and IncFIA(HI1) replicons (Fig. 4). In addition, the contig exhibited 99.81% identity (96% coverage) to plasmid pIncH1 (GenBank accession number CP068510) in Salmonella enterica subsp. *enterica* serovar Derby strain 19CS0402. pIncH1 was an IncHI1B(R27) and IncHI1A plasmid without *mcr-1* (Fig. 4). We mapped the draft genome assemblies of E. coli CP67 against plasmid pCP53-mcr1_3 and found high coverage in the region carrying IncHI1B(R27), IncHI1A, and IncFIA(HI1) replicons (Fig. 4). It is possible that *mcr-1* in CP67 was located on a hybrid plasmid with IncHI1B(R27), IncHI1A, and IncFIA(HI1) replicons or with only IncHI1B(R27) and IncHI1A.

### Analysis of the genetic environment of *mcr-1*.

**FIG 5:**
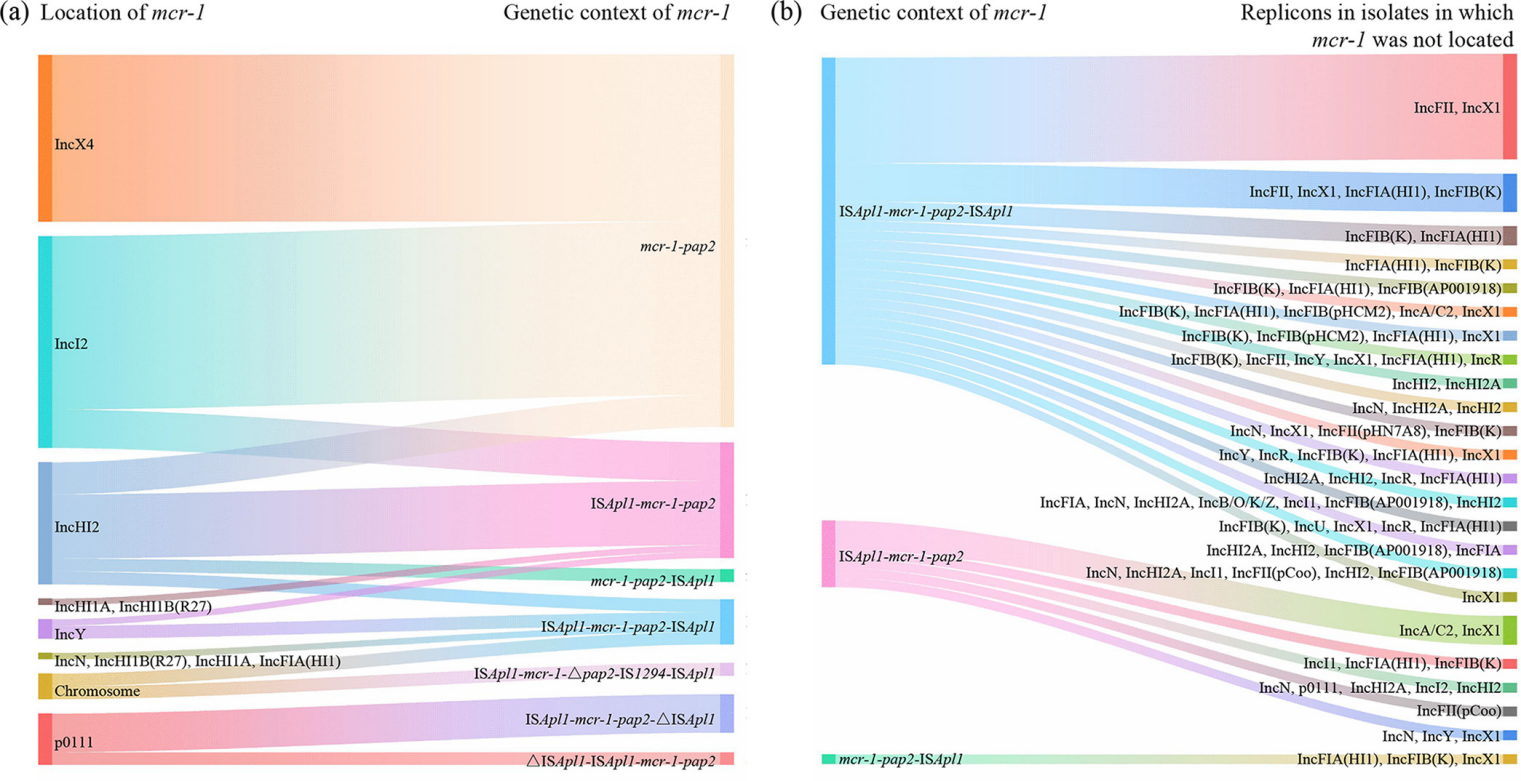
Sankey diagram of the distribution of the genetic context of *mcr-1*. (a) Correlation between the genetic context of *mcr-1* and the types of plasmids where *mcr-1* resides. (b) Genetic context of *mcr-1* in 40 isolates for which the locations of *mcr-1* could not be determined and replicons in these 40 isolates.

The genetic environment of *mcr-1* was explored by analyzing the draft assembly sequences. Seven *mcr-1*-bearing structures were present in 135 *mcr-1*-bearing isolates, including *mcr-1–pap2* (*n* = 58), IS*Apl1–mcr-1–pap2–*IS*Apl1* (*n* = 39), IS*Apl1–mcr-1–pap2* (*n* = 25), IS*Apl1–mcr-1–pap2–*ΔIS*Apl1* (*n* = 6), *mcr-1–pap2–*IS*Apl1* (*n* = 3), IS*Apl1–mcr-1–*Δ*pap2–*IS*1294–*IS*Apl1* (*n* = 2), and ΔIS*Apl1–*IS*Apl1–mcr-1–pap2* (*n* = 2). *mcr-1–pap2* was the dominant *mcr-1*-bearing structure, followed by IS*Apl1–mcr-1–pap2–*IS*Apl1*, which was termed Tn*6330* (20), and IS*Apl1–mcr-1–pap2*. It is worth mentioning that *mcr-1*-bearing structures on IncX4 plasmids were *mcr-1–pap2* (Fig. 5a). Two structures, *mcr-1–pap2* and IS*Apl1–mcr-1–pap2*, were found on IncI2 plasmids, with *mcr-1–pap2* being the predominant structure (Fig. 5a). Four structures, IS*Apl1–mcr-1–pap2*, *mcr-1–pap2*, IS*Apl1–mcr-1–pap2–*IS*Apl1*, and *mcr-1–pap2–*IS*Apl1*, were present in the IncHI2 plasmids, with IS*Apl1–mcr-1–pap2* being the primary structure (Fig. 5a). Two structures, IS*Apl1–mcr-1–pap2–*ΔIS*Apl1* and ΔIS*Apl1–*IS*Apl1–mcr-1–pap2*, were found on p0111 plasmids, with IS*Apl1–mcr-1–pap2–*ΔIS*Apl1* being the predominant structure (Fig. 5a). IS*Apl1–mcr-1–pap2–*IS*Apl1* and IS*Apl1–mcr-1–pap2* were the major structures on IncY and two hybrid plasmids (Fig. 5a).

For the 40 isolates for which the locations of *mcr-1* could not be determined, the *mcr-1*-bearing structures included IS*Apl1–mcr-1–pap2–*IS*Apl1* (*n* = 32), IS*Apl1–mcr-1–pap2* (*n* = 7), and *mcr-1–pap2–*IS*Apl1* (*n* = 1) (Fig. 5b). The plasmid replicons in these isolates were diverse, and we speculated that *mcr-1* may be located on chromosomes, IncHI2, IncY, or hybrid plasmids (Fig. 5b). Among the 60 isolates from which *mcr-1* was transferable, the location of *mcr-1* was not known for 11 isolates (Table S1). Among these 11 isolates, the *mcr-1*-bearing structure in 7 isolates was IS*Apl1–mcr-1–pap2–*IS*Apl1* (Tn*6330*), which could be transferred by itself or by plasmids. The *mcr-1*-bearing structures in the remaining 4 isolates were IS*Apl1–mcr-1–pap2*, which were more likely to be on transferable plasmids (Table S1).

## DISCUSSION

Numerous studies have reported the prevalence and characteristics of *mcr-1*-positive Enterobacteriaceae in China, and E. coli was found to be a critical host of *mcr-1* in both medical and veterinary settings (4). The positivity rate for *mcr-1*-bearing E. coli isolates from pigs in northeastern China (including Heilongjiang, Jilin, and Liaoning) between July 2016 and June 2017 was 28.0% (56/200) (21). Furthermore, the positivity rate for *mcr-1*-carrying porcine E. coli isolates in Henan between June 2016 and February 2017 was 25.5% (78/306) (22). A study on the prevalence of *mcr-1*-bearing E. coli strains in 600 fecal samples collected from pig farms in 18 provinces of China between October 2016 and September 2017 showed that the rate of carriage of *mcr-1* was extremely high (457/600) (76.2% on average, ranging from 45.0% to 100% in different provinces) (23), which was considerably higher than that observed previously. In the current study, we determined that the positivity rate for *mcr-1*-carrying E. coli isolates in pig feces in Sichuan was 54.6% (54/99) in 2016. These investigations showed that the prevalence of *mcr-1*-carrying E. coli varied by sampling region, sampling time, and research method. In general, E. coli carrying *mcr-1* was highly prevalent in China before polymyxin was banned as an animal feed additive. A significant decrease in the prevalence of *mcr-1* was observed after the polymyxin withdrawal policy for animal feed was implemented (16, 17). However, continuous monitoring of polymyxin resistance is essential, particularly with regard to early warning for polymyxin management in hospitals.

In the present study, the prevalence of *mcr-1*-positive E. coli was the highest in pig feces (54.6%), while among retail foods samples, the prevalence of *mcr-1*-positive E. coli was the highest in pork (32.9%). As one of the most important food-producing animals, pigs are reared in proximity to humans around the world. Therefore, *mcr-1*-positive E. coli may be transmitted to humans through the food production chain. The high prevalence of *mcr-1*-positive E. coli in pigs and pork poses a potential threat to public health. Importantly, *mcr-1*-bearing ST10 E. coli was found in pigs, pork, and humans simultaneously, which demonstrates that ST10 E. coli is an important vehicle for the spread of *mcr-1* among animals, foods, and humans. Especially, ST10 E. coli was also the vehicle for other resistance genes such as the tigecycline resistance gene *tet*(X4) (24), the carbapenem resistance gene *bla*_NDM-5_ (25), the fosfomycin resistance gene *fosA3*, and the extended-spectrum-β-lactamase (ESBL)-encoding genes *bla*_OXA_ and *bla*_TEM_ (26).

Plasmids are extrachromosomal DNA elements that can confer new determinants to bacteria for adaptation to new environments (27). *mcr-1*-bearing plasmids markedly contribute to the prevalence of polymyxin resistance. Diverse types of plasmids can mediate the transmission of *mcr-1* in field and clinical isolates, including IncHI2, IncX4, IncI2, IncP, IncY, IncFII, IncHI1, IncFI, IncK2, IncX1, IncFIA, IncFIB, IncR, IncQ1, and hybrid plasmids (IncX1-IncX2, IncI2-IncFIB, and IncX3-IncX4) (20, 28–33). The global distribution of *mcr-1* is mediated mainly by IncHI2, IncX4, and IncI2 plasmids (10, 12). An *mcr-1*-carrying IncHI2 plasmid could impose fitness costs on the host cell, but this cost was largely compensated for after long-term culturing (34). In addition, fitness advantages and high efficiencies of transfer of *mcr-1*-bearing IncI2 and IncX4 plasmids in E. coli were observed (35). This may explain the prevalence of *mcr-1*-harboring IncI2, IncX4, and IncHI2 plasmids globally. In addition, *mcr-1*-bearing IncI2, IncX4, and IncHI2 plasmids are typically conjugative plasmids (12). However, some *mcr-1*-bearing IncI2, IncX4, and IncHI2 plasmids were not transferred to recipients, which warrants further investigation.

Although *mcr-1*-bearing plasmids are diverse, the *mcr-1* gene is frequently accompanied by the IS*Apl1* element, which belongs to the IS*30* family, surrounded by imperfect terminal inverted repeat sequences (36). It has been reported that the *mcr-1* gene can be mobilized by the IS*Apl1*-mediated composite transposon (Tn*6330*) (37). However, *mcr-1–pap2* cassettes lacking IS*Apl1* or possessing one IS*Apl1* element upstream have been commonly identified and were demonstrated to be formed by the deletion of IS*Apl1* from the ancestral Tn*6330* element (38). In the current study, *mcr-1–pap2* (58/135) was the dominant *mcr-1*-bearing structure, followed by IS*Apl1–mcr-1–pap2–*IS*Apl1* (39/135) and IS*Apl1–mcr-1–pap2* (25/135). The *mcr-1*-bearing structures on IncX4 plasmids were only *mcr-1–pap2* cassettes, which was consistent with the results of a previous study suggesting that IS*Apl1* was presumably involved in the transposition of the *mcr-1–pap2* cassette and then was lost (39), and *mcr-1* could be further propagated by conjugative IncX4 plasmids. The remaining four structures were considered derivatives of Tn*6330*. These derivatives may further evolve into *mcr-1–pap2* cassettes and spread *mcr-1* by plasmids. The high prevalence of Tn*6330* may accelerate the transmission of *mcr-1* and represents a significant threat to global public health.

### Conclusion.

In conclusion, IncI2, IncX4, and IncHI2 plasmids were the major vectors for the spread of *mcr-1* from different geographical locations and sources, and the prevalence of Tn*6330* may accelerate the transmission of *mcr-1*. The high prevalence of *mcr-1*-positive E. coli strains in pigs and pork as well as the horizontal transmission of *mcr-1*-bearing plasmids in diverse E. coli strains suggest that pigs and pork are vital sources of *mcr-1*-positive strains in humans and pose a potential threat to public health. Continuous surveillance of *mcr-1* and variants in bacteria after polymyxin withdrawal is essential for evaluating the public health risk posed by *mcr* genes.

## MATERIALS AND METHODS

### Bacterial isolates and identification.

Between July and December 2016, 847 samples, including 99 pig fecal samples, 447 retail food samples (including 155 pork samples, 66 chicken meat samples, and 226 vegetable samples), and 301 patient fecal samples, were collected from Hebei, Henan, and Sichuan Provinces in China. Food samples were enriched in E. coli broth containing 4 μg/mL polymyxin B for 18 h at 37°C and then streaked onto MacConkey agar plates. Fecal samples were directly streaked onto MacConkey agar plates. Typical lactose-fermenting colonies growing as red or pink colonies were purified and subcultured onto tryptic soy agar plates for species identification, which was carried out using a Vitek2 compact automatic microbial identification system, a matrix-assisted laser desorption ionization–time of flight mass spectrometry (MALDI-TOF MS) system, and 16S rRNA gene sequencing. These isolates were subsequently screened for the presence of *mcr-1* by PCR with specific primers (*mcr-1*-F [ATCAGCCAAACCTATCCTATCG] and *mcr-1*-R [ATAGATGTTGCTGTGCGTCTGC]) and Sanger sequencing.

### Antimicrobial susceptibility testing.

The MICs of ampicillin, cefotaxime, ceftazidime, imipenem, gentamicin, trimethoprim-sulfamethoxazole, chloramphenicol, azithromycin, tetracycline, ciprofloxacin, nalidixic acid, and polymyxin B against all *mcr-1*-bearing isolates were determined by the broth microdilution method according to Clinical and Laboratory Standards Institute (CLSI) guidelines (40). MIC breakpoints were interpreted according to CLSI and European Committee on Antimicrobial Susceptibility Testing (EUCAST) (v11.0) (http://www.eucast.org/clinical_breakpoints/) guidelines. E. coli ATCC 25922 served as the quality control strain.

### PFGE and MLST.

PFGE was performed to assess the genetic relatedness of all *mcr-1*-positive isolates according to the PulseNet protocol (https://pulsenetinternational.org/protocols/), using a Chef Mapper system (Bio-Rad, Hercules, CA, USA). Bacterial DNA was digested with XbaI (TaKaRa, Shiga, Japan), and Salmonella enterica serovar Braenderup H9812 restricted with XbaI was used as the reference standard. Cluster analysis of PFGE-XbaI fingerprints was performed using BioNumerics (Applied Maths, bioMérieux, Sint-Martens-Latem, Belgium). MLST was performed by PCR with primers for seven housekeeping genes (*adk*, *fumC*, *gyrB*, *icd*, *mdh*, *purA*, and *recA*). The amplicons were purified and then subjected to Sanger sequencing. The sequencing results were analyzed according to E. coli MLST database guidelines (https://pubmlst.org/bigsdb?db=pubmlst_ecoli_achtman_seqdef).

### Conjugation experiments.

Isolates carrying *mcr-1* were subjected to conjugation assays to study the transferability of *mcr-1*. Briefly, *mcr-1*-positive isolates were used as donors, and E. coli 26R 793 (resistant to rifampin) was used as the recipient. Cultures of donors and the recipient with a culture density of a 0.5 McFarland standard were mixed at a ratio of 1:1, respectively, and the mixtures were then incubated at 37°C statically. After incubation for 12 to 14 h, we subsequently 10-fold serially diluted the mixtures in sterile saline and aliquoted 100 μL of the diluted culture onto selective LB agar plates containing polymyxin B (4 μg/mL) and rifampin (100 μg/mL). The *mcr-1*-positive transconjugants were screened by PCR and polymyxin B resistance phenotypes.

### Genomic DNA sequencing and bioinformatic analysis.

The genomic DNAs of *mcr-1*-harboring isolates were extracted using the FastPure bacterial DNA isolation minikit (Vazyme, Nanjing, China) according to the manufacturer’s recommendations. Whole-genome sequencing was performed on the Illumina HiSeq X ten platform to acquire short-read data. The short-read Illumina raw sequences of *mcr-1*-bearing isolates were separately assembled using SPAdes (41). The plasmid replicons and AMR genes were analyzed using PlasmidFinder and ResFinder (https://www.genomicepidemiology.org/services/). We determined the location of *mcr-1* by analyzing the replicon types on the contigs where *mcr-1* resides. In addition, the sequences coharboring *mcr-1* and replicons could be used as the reference plasmid sequences to understand the localization of *mcr-1* in other bacteria. The core-genome MLST allelic profiles of E. coli were built using phyloviz (42). The phylotyping of E. coli was performed using clermont.py software (https://github.com/A-BN/ClermonTyping). The draft genomes were annotated using Prokka (43). A pangenome analysis was conducted on the *mcr-1*-bearing E. coli isolates using Roary (44). The phylogenetic tree of all *mcr-1*-positive isolates was constructed using FastTree based on SNPs of core genomes (45). The phylogenetic tree was visualized by using iTOL (https://itol.embl.de/). TBtools was used to visualize the distributions of AMR genes (46). BRIG was used to generate a plasmid comparison map (47).

### Data availability.

The whole-genome sequencing data for 129 *mcr-1*-positive isolates can be found in the NCBI database under BioProject accession number PRJNA560609.
