# Supplementary material for: Prevalence and Genomic Characteristics of mcr-Positive Escherichia coli Strains Isolated from Humans, Pigs, and Foods in China
Source: Microbiol Spectr. 2023 Apr 12;11(3):e04569-22. doi: 10.1128/spectrum.04569-22 (PMC10269804; doi:10.1128/spectrum.04569-22)
Supplement: Supplemental file 1 — Supplemental material. Download spectrum.04569-22-s0001.pdf, PDF file, 1.3 MB [file spectrum.04569-22-s0001.pdf]

Supplementary materials

Table S1 The basic characterization of 60 *mcr-1*-bearing isolates in which *mcr-1* was transferable.

Isolates	MLST	Source	Region	Location of *mcr-1*	Genetic context of *mcr-1*	Replicons
CP4	ST10	Pig faeces	Sichuan	IncX4	*mcr-1-pap2*	IncX4, IncR, IncX1
CP9	ST101	Pig faeces	Sichuan	IncHI2	*mcr-1-pap2*	IncHI2A, IncX1, IncR, IncHI2, IncFIB(K), IncFIA(HI1), IncN
CP15	ST1716	Pig faeces	Sichuan	-	IS*Apl1-mcr-1-pap2*-IS*Apl1*	IncFII, IncX1, IncFIA(HI1), IncFIB(K)
CP17	ST10	Pig faeces	Sichuan	-	IS*Apl1-mcr-1-pap2*-IS*Apl1*	IncFIB(K), IncFIA(HI1), IncFIB(pHCM2), IncA/C2, IncX1
CP18	ST1716	Pig faeces	Sichuan	-	IS*Apl1-mcr-1-pap2*	IncA/C2, IncX1
CP20	ST1716	Pig faeces	Sichuan	-	IS*Apl1-mcr-1-pap2*	IncA/C2, IncX1
CP21	ST1716	Pig faeces	Sichuan	-	IS*Apl1-mcr-1-pap2*-IS*Apl1*	IncFII, IncX1
CP27	ST101	Pig faeces	Sichuan	IncI2	*mcr-1-pap2*	IncI1, IncI2, IncFIA, IncFIC(FII), IncFII(pRSB107), IncFIB(AP001918)
CP54	ST101	Pig faeces	Sichuan	IncI2	*mcr-1-pap2*	IncI1, IncI2, IncFIA, IncFIC(FII), IncFII(pRSB107), IncFIB(AP001918)
CP55	ST971	Pig faeces	Sichuan	IncX4	*mcr-1-pap2*	IncB/O/K/Z, IncFIB(AP001918), IncFII, IncX4
CP62	ST5748	Pig faeces	Sichuan	IncX4	*mcr-1-pap2*	IncFIB(AP001918), IncFIC(FII), IncX4
CP63	ST1716	Pig faeces	Sichuan	-	IS*Apl1-mcr-1-pap2*	IncA/C2, IncX1
CP64	ST1716	Pig faeces	Sichuan	-	IS*Apl1-mcr-1-pap2*-IS*Apl1*	IncFII, IncX1
CP66-4	ST165	Pig faeces	Sichuan	IncI2	*mcr-1-pap2*	IncN, IncI2, IncFIA(HI1), IncR, IncX1, IncFIB(K)
CP71	ST1589	Patients feces	Sichuan	IncI2	IS*Apl1-mcr-1-pap2*	IncI1, IncY, IncI2
CP74	ST2705	Patients feces	Sichuan	IncX4	*mcr-1-pap2*	IncI2, p0111, IncX4

CP86	ST1589	Patients feces	Sichuan	IncI2	IS*Apl1-mcr-1-pap2*	IncI1, IncY, IncI2
CP88	ST685	Patients feces	Sichuan	IncX4	*mcr-1-pap2*	IncI1, IncX4
CP92	ST1629	Patients feces	Sichuan	IncX4	*mcr-1-pap2*	IncFIC(FII), IncX4, IncFIB(AP001918)
CP96	ST10	Patients feces	Sichuan	IncX4	*mcr-1-pap2*	IncX4, IncFIB(K)
CP97	ST-	Patients feces	Sichuan	IncI2	IS*Apl1-mcr-1-pap2*	IncFII(pHN7A8), IncFIC(FII), IncX1, IncI2
CP98	ST117	Patients feces	Sichuan	IncI2	IS*Apl1-mcr-1-pap2*	IncI2, IncFIC(FII), IncFIB(AP001918)
CP109	ST196	Pig faeces	Sichuan	IncI2	IS*Apl1-mcr-1-pap2*	IncI2, IncFIC(FII), IncFIB(AP001918), IncFIA
CP113	ST1716	Pig faeces	Sichuan	-	IS*Apl1-mcr-1-pap2*-IS*Apl1*	IncFII, IncX1
CP119	ST46	Pork	Sichuan	IncX4	*mcr-1-pap2*	IncFIB(K), IncX4, IncFII, IncFIA(HI1)
CP123	ST-	Vegetables	Sichuan	IncX4	*mcr-1-pap2*	IncY, IncX4, IncR, IncFIA(HI1), IncFIB(K)
CP132	ST744	Pork	Sichuan	IncX4	*mcr-1-pap2*	IncFIB(AP001918), IncX4, IncFIC(FII)
CP133	ST744	Pork	Sichuan	IncX4	*mcr-1-pap2*	IncFIB(AP001918), IncX4, IncFIC(FII)
CP134	ST10	Vegetables	Sichuan	IncX4	*mcr-1-pap2*	IncFII(pHN7A8), IncX4, IncFIB(K), IncFIA(HI1)
CP137	ST542	Pork	Sichuan	IncX4	*mcr-1-pap2*	IncFIB(K), IncX4, IncFIB(pHCM2)
CP139	ST6438	Pork	Sichuan	IncX4	*mcr-1-pap2*	IncX1, IncFIB(AP001918), IncX4, IncFIC(FII), IncFIB(K), IncFIA(HI1)
CP142	ST-	Pork	Sichuan	IncX4	*mcr-1-pap2*	IncP1, IncX4, IncN, IncR, IncX3
CP144	ST6438	Pork	Sichuan	IncX4	*mcr-1-pap2*	IncFIA, IncN, IncY, IncFIC(FII), IncFIB(AP001918), IncI1, IncX4, IncX1
HP16	ST206	Pork	Henan	IncX4	*mcr-1-pap2*	IncFIB(K), IncX4, IncX1, IncFIA(HI1), IncFII
HP25	ST3941	Chicken	Henan	IncI2	*mcr-1-pap2*	IncI2, IncI1, IncFIA, IncFIB(AP001918)
HP33	ST2345	Pork	Henan	IncX4	*mcr-1-pap2*	IncX4, IncFIA(HI1), IncFIB(K)
HP34	ST3107	Chicken	Henan	IncI2	*mcr-1-pap2*	IncHI2, IncHI2A, IncFIC(FII), IncI2, IncI1, IncFIB(AP001918)
HP37	ST2973	Chicken	Henan	IncI2	*mcr-1-pap2*	IncN, IncY, IncI2, IncFIB(AP001918), IncFIC(FII), IncFII(pHN7A8), IncX1
HP45	ST2973	Chicken	Henan	IncI2	*mcr-1-pap2*	p0111, IncFIB(pHCM2), IncI1, IncI2
HP51	ST10	Pork	Henan	IncX4	*mcr-1-pap2*	IncX4
HP57	ST46	Pork	Henan	IncX4	*mcr-1-pap2*	IncX1, IncX4, IncFIB(AP001918), IncFIC(FII)

HP59	ST10	Pork	Henan	IncX4	*mcr-1-pap2*	IncFIC(FII), IncY, IncX4, IncFIB(AP001918)
HP79	ST46	Patients feces	Henan	IncI2	*mcr-1-pap2*	IncI2
SJP1	ST23	Pork	Hebei	IncI2	IS*Apl1-mcr-1-pap2*	IncI2, IncFIC(FII), IncFIB(AP001918), IncB/O/K/Z
SJP45	ST602	Pork	Hebei	IncI2	*mcr-1-pap2*	IncI2, IncFIB(AP001918), IncI, IncFII(pHN7A8)
SJP88	ST170	Pork	Hebei	IncI2	*mcr-1-pap2*	IncHI1B(CIT), IncI2, IncFIB(pHCM2), p0111, IncFIB(K)
SJP90	ST-	Pork	Hebei	IncI2	*mcr-1-pap2*	IncHI1B(CIT), IncI1, IncI2, IncFII(pHN7A8), p0111, IncFIB(K)
SJP113	ST93	Patients feces	Hebei	IncI2	*mcr-1-pap2*	IncFIB(AP001918), IncY, IncB/O/K/Z, IncA/C2, IncI2
SJP114	ST4204	Pork	Hebei	-	IS*Apl1-mcr-1-pap2*-IS*Apl1*	IncHI2A, IncHI2, IncR, IncFIA(HI1)
SJP120	ST744	Pork	Hebei	IncX4	*mcr-1-pap2*	IncX4, IncX1, IncFIA(HI1), IncFIB(K)
SJP121	ST226	Pork	Hebei	-	IS*Apl1-mcr-1-pap2*	IncN, p0111, IncHI2A, IncI2, IncHI2
SJP128	ST209	Pork	Hebei	IncI2	*mcr-1-pap2*	IncN, IncI2, IncFIB(AP001918), IncFIC(FII), IncFII(pHN7A8)
SJP130	ST-	Pork	Hebei	IncI2	*mcr-1-pap2*	IncI2, p0111, IncFIB(AP001918), IncI
SJP136	ST10	Pork	Hebei	IncX4	*mcr-1-pap2*	IncX4
SJP170	ST7315	Pork	Hebei	IncI2	*mcr-1-pap2*	IncN, IncI2, IncFIB(K), IncHI2A, IncHI2
SJP174	ST2253	Pork	Hebei	IncI2	*mcr-1-pap2*	IncFIC(FII), IncB/O/K/Z, IncI2, IncFIC(FII), IncFIB(AP001918)
SJP178	ST155	Pork	Hebei	IncI2	*mcr-1-pap2*	IncI2, IncFIC(FII), IncR
SJP179	ST58	Pork	Hebei	-	IS*Apl1-mcr-1-pap2*-IS*Apl1*	IncN, IncHI2A, IncI1, IncFII(pCoo), IncHI2, IncFIB(AP001918),
SJP181	ST457	Pork	Hebei	IncI2	*mcr-1-pap2*	IncI1, IncI2, IncR, IncX1, IncFIB(AP001918)
SJP182B	ST744	Pork	Hebei	IncI2	*mcr-1-pap2*	IncI2, IncFIC(FII), IncFIB(AP001918)

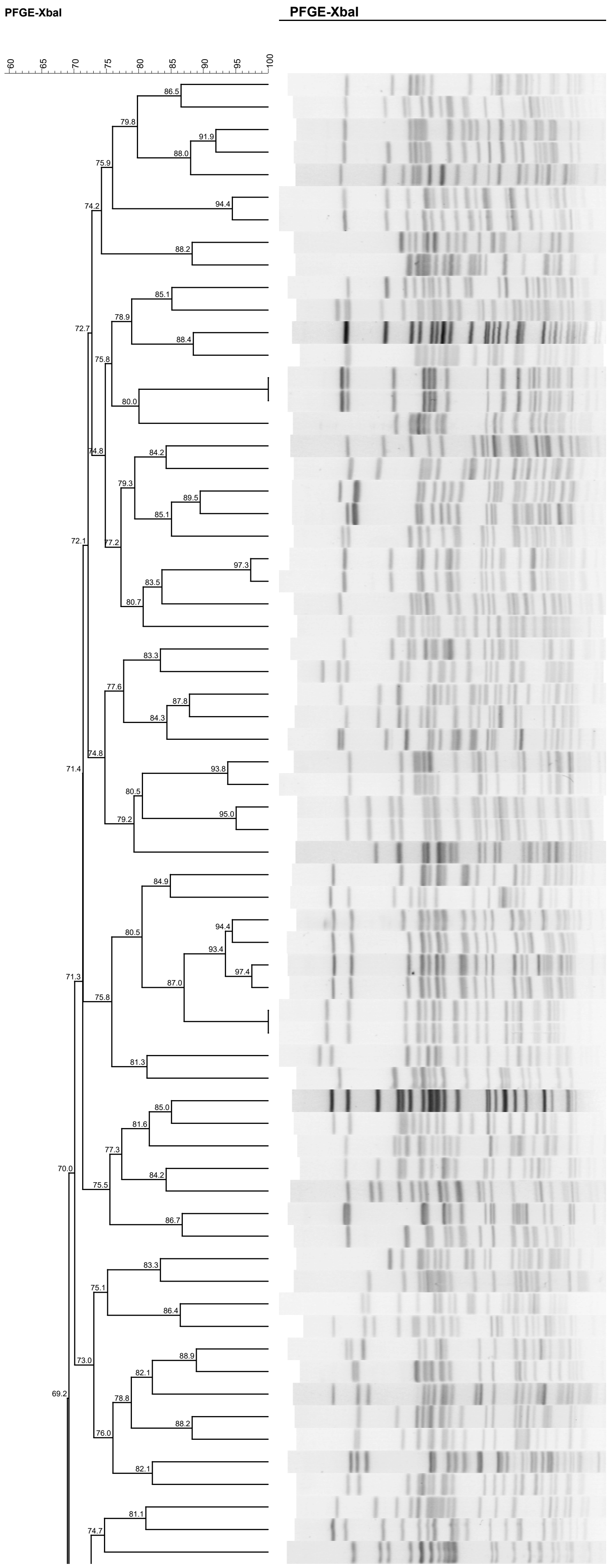

CP 68	Sichuan	pig faeces	ST4015
CP 119	Sichuan	Pork	ST46
CP 145	Sichuan	Pork	ST871
CP 138	Sichuan	Pork	ST871
SJP 170	Hebei	Pork	ST7315
CP 98	Sichuan	Children	ST117
CP 42	Sichuan	Vegetables	ST1684
CP 65	Sichuan	pig faeces	ST101
CP 66-6	Sichuan	pig faeces	ST5229
CP 2	Sichuan	pig faeces	ST189
CP 142	Sichuan	Pork	ST10
CP 131	Sichuan	pig faeces	ST48
SJP 89	Hebei	Pork	ST10
CP 9	Sichuan	pig faeces	ST101
CP 10	Sichuan	pig faeces	ST101
SJP 174	Hebei	Pork	ST2253
SJP 142	Hebei	Pork	ND
HP 70	Henan	Children	ST1249
CP 23	Sichuan	pig faeces	ST10
CP 17	Sichuan	pig faeces	ST10
CP 134	Sichuan	Vegetables	ST10
CP 45	Sichuan	Children	ST117
CP 41	Sichuan	Pork	ST1684
HP 59	Henan	Pork	ST10
SJP 45	Hebei	Pork	ST602
CP 80	Sichuan	Human	ST799
SJP 88	Hebei	Pork	ST170
SJP 130	Hebei	Pork	ST156
SJP 179	Hebei	Pork	ST58
CP 11	Sichuan	pig faeces	ST165
CP 54	Sichuan	pig faeces	ST101
CP 27	Sichuan	pig faeces	ST101
SJP 18	Hebei	Pork	ST101
HP 83	Henan	Children	ST101
SJP 155	Hebei	Pork	ST5912
CP 26	Sichuan	pig faeces	ST4429
CP 92	Sichuan	Children	ST1629
CP 127	Sichuan	pig faeces	ST4429
CP 14	Sichuan	pig faeces	ST4429
CP 5	Sichuan	pig faeces	ST4429
CP 16	Sichuan	pig faeces	ST4429
CP 111	Sichuan	pig faeces	ST4429
CP 112	Sichuan	pig faeces	ST4429
CP 107	Sichuan	pig faeces	ST48
CP 120	Sichuan	Pork	ST101
CP 55	Sichuan	pig faeces	ST971
HP 78	Henan	Chicken	ST48
SJP 128	Hebei	Pork	ST209
SJP 119	Hebei	Pork	ST48
SJP 140	Hebei	Pork	ST609
CP 4	Sichuan	pig faeces	ST10
CP 137	Sichuan	Pork	ST542
CP 128	Sichuan	pig faeces	ST165
HP 63	Henan	Pork	ST744
CP 116	Sichuan	pig faeces	ST48
SJP 113	Hebei	Human	ST93
CP 70	Sichuan	pig faeces	ST1716
SJP 120	Hebei	Pork	ST744
SJP 173	Hebei	Pork	ST744
SJP 182B	Hebei	Pork	ST744
CP 133	Sichuan	Pork	ST744
CP 7	Sichuan	pig faeces	ST2035
CP 132	Sichuan	Pork	ST744
CP 129	Sichuan	pig faeces	ST224
SJP 178	Hebei	Pork	ST155
CP 8-4	Sichuan	pig faeces	ST2614

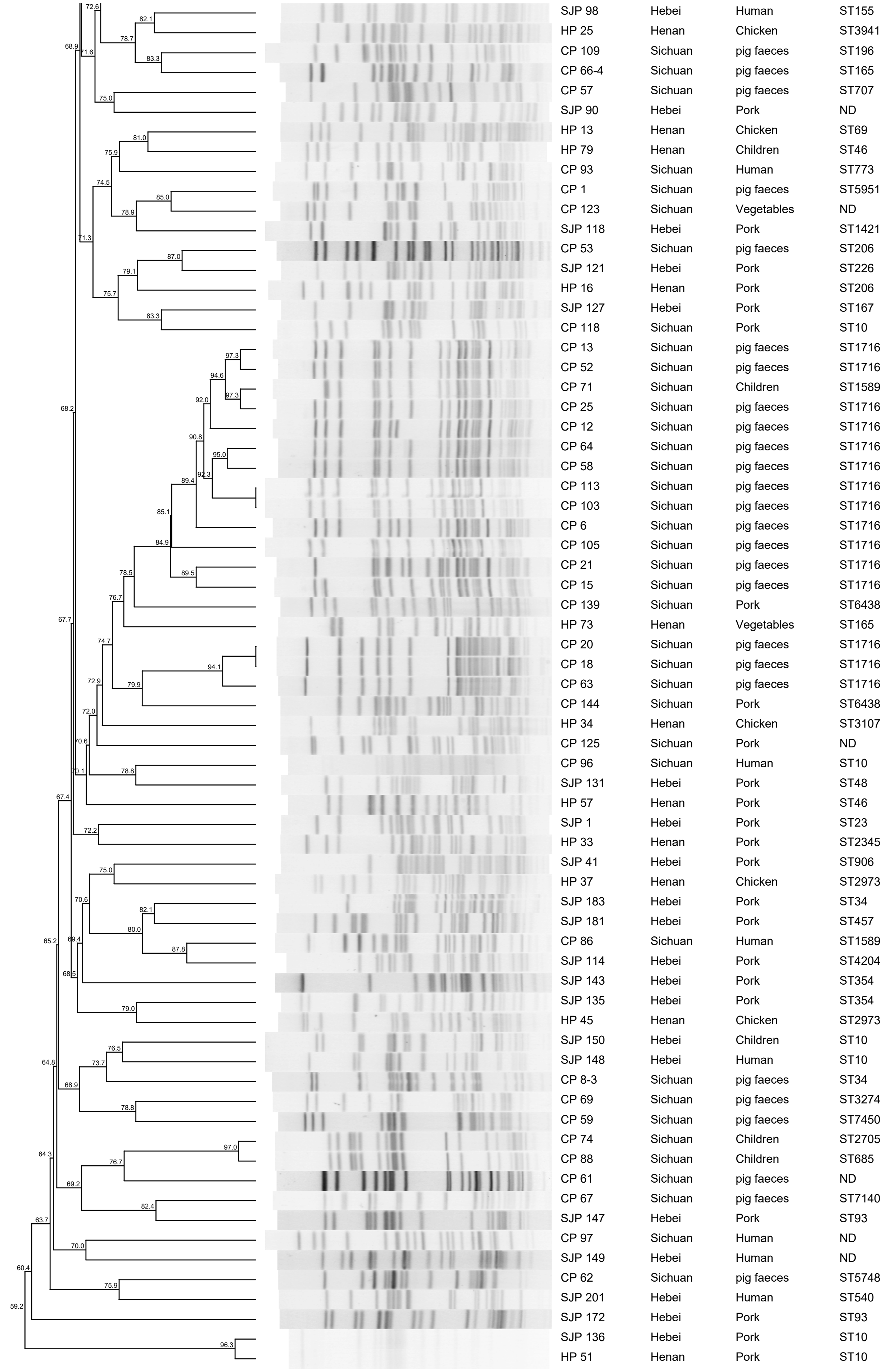
SJP 98 Hebei Human ST155
HP 25 Henan Chicken ST3941
CP 109 Sichuan pig faeces ST196
CP 66-4 Sichuan pig faeces ST165
CP 57 Sichuan pig faeces ST707
SJP 90 Hebei Pork ND
HP 13 Henan Chicken ST69
HP 79 Henan Children ST46
CP 93 Sichuan Human ST773
CP 1 Sichuan pig faeces ST5951
CP 123 Sichuan Vegetables ND
SJP 118 Hebei Pork ST1421
CP 53 Sichuan pig faeces ST206
SJP 121 Hebei Pork ST226
HP 16 Henan Pork ST206
SJP 127 Hebei Pork ST167
CP 118 Sichuan Pork ST10
CP 13 Sichuan pig faeces ST1716
CP 52 Sichuan pig faeces ST1716
CP 71 Sichuan Children ST1589
CP 25 Sichuan pig faeces ST1716
CP 12 Sichuan pig faeces ST1716
CP 64 Sichuan pig faeces ST1716
CP 58 Sichuan pig faeces ST1716
CP 113 Sichuan pig faeces ST1716
CP 103 Sichuan pig faeces ST1716
CP 6 Sichuan pig faeces ST1716
CP 105 Sichuan pig faeces ST1716
CP 21 Sichuan pig faeces ST1716
CP 15 Sichuan pig faeces ST1716
CP 139 Sichuan Pork ST6438
HP 73 Henan Vegetables ST165
CP 20 Sichuan pig faeces ST1716
CP 18 Sichuan pig faeces ST1716
CP 63 Sichuan pig faeces ST1716
CP 144 Sichuan Pork ST6438
HP 34 Henan Chicken ST3107
CP 125 Sichuan Pork ND
CP 96 Sichuan Human ST10
SJP 131 Hebei Pork ST48
HP 57 Henan Pork ST46
SJP 1 Hebei Pork ST23
HP 33 Henan Pork ST2345
SJP 41 Hebei Pork ST906
HP 37 Henan Chicken ST2973
SJP 183 Hebei Pork ST34
SJP 181 Hebei Pork ST457
CP 86 Sichuan Human ST1589
SJP 114 Hebei Pork ST4204
SJP 143 Hebei Pork ST354
SJP 135 Hebei Pork ST354
HP 45 Henan Chicken ST2973
SJP 150 Hebei Children ST10
SJP 148 Hebei Human ST10
CP 8-3 Sichuan pig faeces ST34
CP 69 Sichuan pig faeces ST3274
CP 59 Sichuan pig faeces ST7450
CP 74 Sichuan Children ST2705
CP 88 Sichuan Children ST685
CP 61 Sichuan pig faeces ND
CP 67 Sichuan pig faeces ST7140
SJP 147 Hebei Pork ST93
CP 97 Sichuan Human ND
SJP 149 Hebei Human ND
CP 62 Sichuan pig faeces ST5748
SJP 201 Hebei Human ST540
SJP 172 Hebei Pork ST93
SJP 136 Hebei Pork ST10
HP 51 Henan Pork ST10

Figure S1 PFGE-*Xba*I dendrogram and details of *mcr-1*-positive *E. coli* isolates. The PFGE assay was conducted according to the standard protocol. The information including sampling site, source, and ST-type were displayed in this figure.
